# Synthetic Evaluation of China’s Regional Low-Carbon Economy Challenges by Driver-Pressure-State-Impact-Response Model

**DOI:** 10.3390/ijerph17155463

**Published:** 2020-07-29

**Authors:** Wenyan Pan, Muhammad Awais Gulzar, Waseem Hassan

**Affiliations:** 1 School of Safety Science and Emergency Management, Wuhan University of Technology, Wuhan 430070, China; panwenyan@whut.edu.cn; 2 University of Waikato Joint Institute, Zhejiang University City College, Hangzhou 310015, China; 3 Waikato Management School, University of Waikato, Hamilton 3240, New Zealand; 4 NUST Business School, National University of Sciences and Technology, Islamabad 44000, Pakistan; waseem.hassan@nbs.nust.edu.pk

**Keywords:** CO_2_ emissions, low-carbon economy, DPSIR model, coupling coordination model

## Abstract

The “driver–pressure–state–impact–response” (DPSIR) model has recently become a popular approach to deal with environmental problems. The combination of DPSIR with analytic hierarchy process (AHP) is a useful method to study low-carbon evaluation because the AHP model has a special advantage in multi-indexes evaluation. This paper constructs the low-carbon economy evaluation system and comprehensively evaluates the numerical value of low-carbon economic development of China’s 30 regions from 2000 to 2015 by using the AHP method. It shows that the numerical value of low-carbon economy of China’s 30 regions varies in terms of growth rate. The numerical value of east regional low-carbon economy shows a pattern that is gradually higher than that of the west region. The numerical value of low carbon economic development in the south region is higher than that of the north region by degrees. In addition, based on the model of coordination degree in 2015, the result indicated that the four subsystems have primary coordination in the east area and bare coordination in the central and west areas. It is indicated that the four sub-indexes should be developed at the same pace and promoting the development of a low-carbon economy in the mid-west areas is the key in China. Finally, we proposed that environmental regulations and policies should be formulated to improve coordination in various aspects and various departments. Calculating the degree of low-carbon economic coupling coordination may be helpful for policy makers to formulate effective policies and take actions in the future.

## 1. Introduction

The 26th meeting of the Conference of the Parties (COP26) of the United Nations Framework Convention on Climate Change (UNFCCC), originally scheduled for November in Glasgow, United Kingdom has been postponed until 2021 [1]. Patricia Espinosa (Executive Secretary of UNFCCC) said COVID-19 is the most urgent threat facing humanity today, but we cannot forget that climate change is the biggest threat over the long term. It clarifies the content of anthropogenic climate change and the political statements of reducing greenhouse gas emissions of each country. An important contribution made by the Paris Agreement (COP21) was the Nationally Determined Contributions (NDCs) [2]. This is the key to achieving long-term goals. The NDCs identified the efforts and commitments of each country to reduce greenhouse gas emissions, improving the impact of climate change. The aim is to work together to respond to the challenge of climate change and the greenhouse effect. The Paris Agreement stipulates that global warming needs to be limited between 1.5 and 2 °C above pre-industrial levels. The European Union (EU) and China as the largest economies and emitters have announced that they will strengthen their collaboration and step-up their efforts to deal with climate change [3]. Greenhouse gas emissions are a very serious environmental problem that the planet is facing today, and it has become a nightmare for countries throughout the world. This issue is mainly attributed to the typical high carbon economy. It is extremely important for the countries around the globe to deal with climate change and achieve a low-carbon economy. In order to explore the corresponding environmental protection measures, the calculation of carbon dioxide emissions and evaluation of low-carbon economic development at the regional level is of great theoretical and practical value [4,5,6,7].

The core objective/essence of low-carbon economy is to reduce CO_2_ emissions. For the purpose of dealing with the impacts of climatic change on the global economy and human existence, the emission of CO_2_ in production and consumption must be strictly controlled [8,9,10]. It is indicated that CO_2_ emissions from burning fossil fuels had increased 49% from the year 1990 to the year 2008 [2]. According to the statistics given by the International Energy Agency [11], China became the world’s largest carbon dioxide emitter and the largest energy consumer [12,13]. All these statistics show that as the global climate problem is becoming more and more serious, the low-carbon economy has become a global hotspot all around the world [1]. In decoupling theory, we know that the amount of materials consumed grows with the economy at the beginning of industrialization. However, this situation will reverse at a certain stage in future, which will realize the growth of economy while decreasing consumption of materials. The meaning on low-carbon economy varies with the places. Different paths and choices should be adopted for CO_2_ emission reduction to areas that are at different stages of development. For example, the developed countries that have achieved industrialization and high human development must be able to achieve absolute reduction of CO_2_ emissions. However, in the developing countries that are still in the process of industrialization, due to their booming population and unachieved basic goals of human development, CO_2_ emissions will continue to grow. Therefore, if they ensure social development along with constant economic growth, the relative reduction of CO_2_ emissions should be regarded as low-carbon economy. Due to different developmental stages in different regions, the problems they are facing vary greatly. For all these reasons, constructing the low-carbon economy evaluation index system as well as finding specific problems and solutions are of great importance. 

The low-carbon economy, which is the outcome of constricting emission requirements and increasing human development at a good level is kind of an economic foundation. The reason we develop a low-carbon economy is to control emissions of CO_2_, which is a global shared vision [14,15]. Therefore, the low-carbon economy should be at the base of the development of production, environmental protection and resource optimization. A thriving low-carbon economy can be developed only when these factors proceed simultaneously, along with the unification of economy, ecosystem and social benefit. Besides, while we are researching the low-carbon economy, we need to integrate resources science, environmental science and the social sciences effectively. For this purpose, an instructional method is required that can not only specify complex problems but also effectively combine each part. As a result, the DPSIR model serves as a good research tool for the low-carbon economic evaluation. 

We aimed to build a comprehensive low-carbon economic system by combining two models, DPSIR and AHP, so as to give a numerical value of the low-carbon economic development of China’s 30 regions from 2000 to 2015. In addition, the spatial distribution was analyzed in the four aspects: “pressure”, “state”, “effect” and “response”. The study largely discussed the applicability of DPSIR model to evaluate China’s regional low-carbon economic challenges; a conceptual model was provided, establishing indicator system–data processing–analysis–making decisions.

## 2. Theoretical Framework

The DPSIR model is considered as a fundamental and effective tool to thoroughly observe the causation between human activities and the natural environment [16,17]. In the late 1980s, the OECD proposed a Pressure–State–Response (PSR) frame model [18]. On that basis, the United Nations proposed the Driving Force–State–Response frame model. Finally, the European Environment Agency and others combined the benefits of the previous two index systems and proposed a new conceptual frame model: Driving Force-Pressure–State–Impact–Response (DPSIR) [19,20]. It can be seen that the DPSIR model is the expansion and revision of PSR model, which adds the “Driving Force” factor as the cause of the “Pressure” factor and the “Impact” force caused by the environment [21]. The scholars who use the model think that it is people’s activities such as production and consumption that put the natural environment under pressure, consequently leading to change in the natural environment. However, we should respond to the effects that the climate change brings. The DPSIR model is widely used for analyzing problems about resources, environment, economy, and society. For instance, Maxim et al. supplemented the DPSIR model by using the complex system theory, and then, the model was used to analyze the risk of biological diversity in the aspects of policy, society and economy [22]; Ness et al. think the DPSIR model is quite up to the requirements of sustainable Scientific Outlook on Development, and with the help of that, they can research the eutrophication in the Baltic Sea [23]. Atkins et al. create a model which supports marine environment decision by integrating the DPSIR model with features of ecosystem service and social welfare [24]. The DPSIR model was selected due to its simplicity and because it is the most powerful communication tool between environment, economy and society [25,26].

From these aforementioned examples, it is clear that the applicability of DSPIR model is very high and that’s why it is used by many scholars for the analysis of all kinds of resources and in environmental as well as social and economic problems. DPSIR model particularly focuses on economic operations, their effects on the environment and their interrelations. Thus, it is a very systematic, comprehensive, widespread and flexible model.

The “Driving force” indicates the potential cause of the changes in environmental conditions, which mainly refers to the development trend of economic activities, social activities and changes in the industrial structure. The “Pressure” is the influence of human activities on natural environment, energy and resources. The “State” refers to the situation of environment under the above-mentioned pressures and mainly considers regional resource consumption and environment degradation. The “Impact” is the influence of system’s status on environment and social economic structure. Lastly, the “Response” shows the methods and effective policies that people create when facing environmental impact.

**Figure 1:**
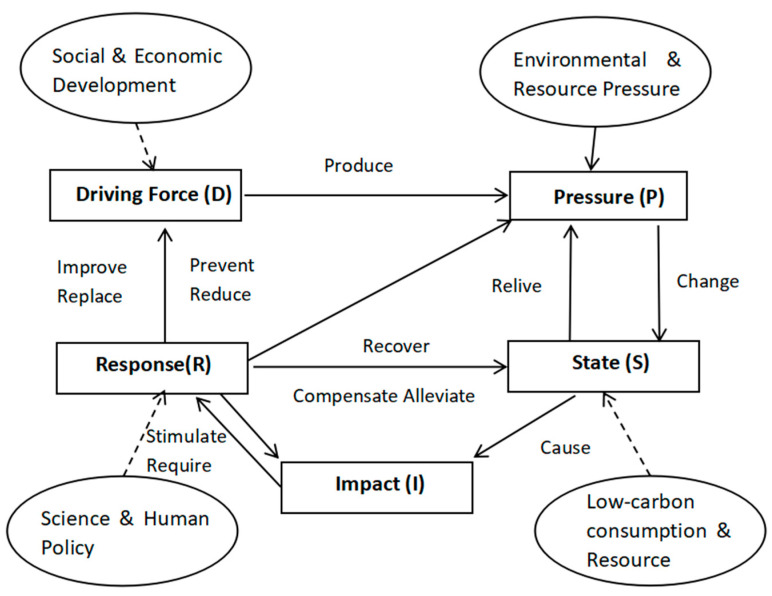
The analytical framework of low-carbon economic system based on driver–pressure–state–impact–response (DPSIR) model.

Firstly, the analytical framework of a low-carbon economy system is constructed, in which the DPSIR model is used considering human needs, social progress, economic development, energy demand, carbon emissions, resource status, low-carbon consumption and economic development. This progress reflects the features of a low-carbon economy, as a complex system relevant to the mutual effect of human activities and natural resources. Showing the connotation of a scientific outlook on development, the low-carbon economic system should be human-oriented and development-coordinated. The analytical framework of the system based on DPSIR model is shown in Figure 1.

In order to construct a multi-dimensional and scientific evaluation index system that not only can conduct the horizontal comparison in all regions but also the vertical reflection of steady shift, the principles mentioned below must be followed.

For the purpose of ensuring that the evaluation of the system completely and objectively reflects its comprehensiveness and shift result, plenty of work needs to be done. In addition to that, the overlap between indexes must be avoided. Therefore, the index system must be made clear by level diversification and index classification according to system structure.

The evaluation index should also scientifically and moderately reveal the properties and transitional characteristics of a low-carbon economy. Besides, every evaluation with its calculation should be standardized, normalized and clearly defined; even those statistics that cannot be obtained from the existing statistical source can be brought into the index system if they have the ability to reflect the practicality and the embodiment of the low-carbon economy.

As a whole, the system should include various indexes that can affect the level of the low-carbon economy. Although it is impossible to cover all the relevant indexes, the system must be capable of reflecting all aspects that can affect the level of the low-carbon economy in the current social economy. In addition, the selected indexes should reflect the main character and status of the evaluated system from different aspects. In order to make the index system easy to use, while choosing indexes, we must emphasize on their depiction and avoid redundancy.

The worldwide recognized common index must be followed after altering it according to required standards, and concurrently, the internationally accepted names, concepts and calculations must be adopted. In addition to that, whatever the evaluation index, system construct is a global perspective oriented job. Therefore, we must take full account of the dynamic change of the system when designing the index system and embrace innovation. 

In this paper, the system is established based on DPSIR model, as shown in Table 1. The “impact” was removed from the DPSIR model because indicators in “impact” overlap with those in “Pressure” and “State”. At the same time, the relevant statistics for economic losses and natural hazard caused by air pollution are hard to get.

The connotation of “Driver” is reflected by setting the “Driver for social development” (D1) and “Driver for economic development” (D2), completely representing the essence of people’s needs and social development of Scientific Outlook on Development. The connotation of “Pressure” is reflected by setting the “resource pressure” (P1) and “environmental pressure” (P2), symbolizing the damages and losses that social progress and human development do to the environment and resources. The connotation of “State” is reflected by setting the “State of low-carbon consumption” (S1) and “State of low-carbon resources” (S2), describing the objective condition of resources and environment in the low-carbon economy system. The connotation of “Response” is reflected by setting the “Scientific Response” (R1), “Human Response” (R2) and “Policy Response” (R3), embodying the positive response of countries towards environmental pollution, resources consumption and ecological destruction. At the same time, the frequently used indexes from the relevant studies about low-carbon economy are selected.

## 3. Methodology

This paper focuses on the coordination degree among four sub-indexes. Degrees of interaction among the four sub-indexes are defined by their respective characteristics as the degree of coupling coordination. The equation is as follows:

(1) $$T=\mu d(x)+\phi p(y)+\xi s(z)+\theta p(k)=\mu \sum_{i=1}^{m}\alpha_{i}x_{i}^{,}+\phi \sum_{i=1}^{n}\beta_{i}y_{i}^{,}+\xi \sum_{i=1}^{j}\sigma_{i}z_{i}^{,}+\theta \sum_{i=1}^{l}\omega_{i}k_{i}^{,}$$

where *T* represents the development degree of the comprehensive evaluation index, which is the reflection of the development level of low-carbon economy. $x_{i}^{,}$ represents the “Driver” indexes. $y_{i}^{,}$ represents “Pressure” indexes. $z_{i}^{,}$ represents “State” indexes. $k_{i}^{,}$ represents “Response” indexes. The weight values are μ, ϕ, ξ, θ, $\alpha_{i}$, $\beta_{i}$, $\sigma_{i}$.

(2) C={d(x)×p(y)×s(z)×p(k)[d(x)+p(y)+s(z)+p(k)4]4}14

where *C* represents the coupling degree, which is a measure of coordinated development. *C* ranges from 0 to 1. The greater the coupling degree among the systems the closer the *C* is to 1. Contrary to it, the smaller the coupling degree among the systems, the closer the *C* is to 0. Besides, there is no need to develop it while the sub-indexes are irrelevant.

(3) $$D=\sqrt{C\times T}$$

*D* is coupling coordinated degree. *T* is development degree. It measures the situation as a whole of the development of coordination in the system [27].

The classification criterion of *D* is as in Table 2.

## 4. Data and Variables

### 4.1. Measurement of CO_2_ Emissions from Energy Consumption

In the current literature and research, the calculation caliber of regional carbon emissions data is difficult to unify. We use the methodology in the IPCC guidelines:

(4) $$C=\sum_{i}T_{i}\times F_{i}$$

where *C* represents total CO_2_ emissions from regional energy consumption; *i* is the type of energy; $T_{i}$ is the terminal consumption for *i*; $F_{i}$ is the coefficient of CO_2_ emissions for *i*:

(5) $$F_{i}=A_{i}\times B_{i}\times (44/12)\times 10^{3}\times 4186.8\times 10^{-9}\times 10^{-3}\times E_{i}$$

$A_{i}$ is the coefficient of carbon emissions for *i* (kgC/GJ); $B_{i}$ is the oxidation rate in the combustion for *i* type energy; $A_{i}$ and $B_{i}$ are derived from IPCC (2006). In Table 3, $C_{i}$ is the coefficient of CO_2_ emissions for *i* (kgCO^2^/TJ); $D_{i}$ is the original coefficient for i type of energy (Kg CO_2_/Kcal); $E_{i}$ is the calorific value of unit fuel in China.

This paper calculates CO_2_ emissions in China’s 30 regions (1995–2016). The data for these provinces are derived from China’s Statistical yearbook. The reason we removed the refinery gas and liquefied petroleum gas is because they are difficult to gather, and they are used less. We calculated the total CO_2_ emissions from 1995 to 2016, which show a tendency to increase year after year (Table 4).

### 4.2. Other Data and Variables

We evaluate the level of the low-carbon economy development level in China’s 30 regions from 2000 to 2015 as shown in Table 1. The demographic data are obtained from “China Population Statistic Yearbook”, the per capita GDP, the GDP growth and the average income of rural and urban families are obtained from the statistics in “China Statistical Yearbook”. The energy data are obtained through the calculation of statistics in “China Energy Statistical Yearbook” and China’s 30 regions’ statistical yearbooks. The scientific data are attained from “China Statistical Yearbook on Science and Technology”. We can set the 6‰ and 75% as moderate indexes, which is the ideal value of natural population growth rate and urbanization rate.

## 5. Regional Evaluation of the Low-Carbon Economy

### 5.1. Index Calculation

Urbanization rate (D12). Urbanization rate refers to the percentage that the urban population accounts for the entire population, which witnesses city’s history and development. Since the current statistics do not cover all sub-provincial cities’ statistical data of urbanization rate, this paper adopts the method that “non-agricultural population accounts for total population proportion” to calculate the urbanization rate, which is authorized by police departments. The method can be summarized as “the urbanization rate = non-agricultural population/total population.

Per capita carbon emissions (S11). From 1990s to the year 2000, the global average annual CO_2_ emission has increased from 22 billion tons to 264 billion tons. Therefore, the primary goal of developing the low-carbon economy is to limit CO_2_ emissions. Due to unavailability of authorized statistics on carbon emissions, this paper estimates the carbon emissions produced by fossil resources (coal, oil and natural gas) based on other existing statistics. The total carbon emissions = Σ (each resource consumption × carbon emissions per unit of energy consumption), summarized as per capita carbon emissions = total carbon emissions/population.

Carbon emissions of residents’ consumption (S12) and government’s consumption (S13). They refer to the carbon emissions created by government’s and residents’ final consumption on goods and services over a period of time, which can reflect the influence of consumption level and structure on carbon emissions. The government’s consumption on carbon emissions refers to the carbon emissions created by government’s consumption on public services, which can reflect the impact of local government governance level on carbon emissions. We account these two indexes according to the proportion of residents’ consumption in GDP (known as final consumption rate) and carbon intensity per unit of economy. That is, carbon emissions of residents’ consumption = total carbon emissions/residents’ consumption, carbon emissions of government’s consumption = total carbon emissions/government’s consumption.

The efficiency of energy process and conversion (R12). The process of energy conversion is a production link of the energy process system, which is the important index for evaluating the advancement of the energy conversion device, the production technique and the quality of administration. The improvement of efficiency of energy process and conversion means that the low energy input can create high energy output. Its formula is as follows: efficiency of energy process and conversion = energy output/energy input.

Carbon productivity (R13). It is considered as the core index of measuring low carbonization, which is the GDP per unit of carbon emitted. This index links the GDP output with the carbon emissions of energy consumption directly. Therefore, it can visually reflect the carbon use efficiency of the whole social economy and measure the overall level of low-carbon technology. In addition, as a result of its relation with economic structure, the carbon productivity can have the potential of further reduction of carbon emissions intensity of unit consumption when the country’s technology has accumulated to a certain extent. Its formula is carbon productivity = GDP/carbon emissions.

### 5.2. Dimensionless Evaluation Factors

The collected original data must be dimensionless through range conversion as they cannot be compared directly.

We set $x_{ki}$ as the value of the index *k* that has been normalized in the evaluated area *i*, and set the $v_{ki}$ as the value of the index *k* in the evaluated area *i*, the *n* as the quantity of the evaluated area. Positive data was calculated:

(6) $$x_{ki}=\frac{v_{ki}-\underset{1\leq i\leq n}{\min}(v_{ki})}{\underset{1\leq i\leq n}{\max}(v_{ki})-\underset{1\leq i\leq n}{\min}(v_{ki})}$$

Negative data was calculated:

(7) $$x_{ki}=\frac{\underset{1\leq i\leq n}{\max}(v_{ki})-v_{ki}}{\underset{1\leq i\leq n}{\max}(v_{ki})-\underset{1\leq i\leq n}{\min}(v_{ki})}$$

Regarding, moderate interrelation for those factors, the closer it approached to ideal value, the better. In reference to a large number of previous literature, this paper sets the planning objective of the country’s natural population growth rate in China (V1 = 6‰) as the ideal value of natural population growth rate (D11) and sets the average urbanization rate in developed countries (V2 = 75%) as the ideal value of the urbanization rate (D12). We set $x_{ki}$ as the value of the index *k* that has been normalized in the evaluated area *i*, set the $v_{ki}$ as the value of the index *k* in the evaluated area *i*, the $v_{ki}^{0}$ as the ideal value and the *n* as the quantity of the evaluated area. Moderate data was calculated:

(8) $$x_{ki}=\{\begin{array}{c} 1-\frac{v_{ki}^{0}-v_{ki}}{\max\left[v_{ki}^{0}-\underset{1\leq i\leq n}{\min}(v_{ki}),\underset{1\leq i\leq n}{\max}(v_{ki})-v_{ki}^{0}\right]},\underset{1\leq i\leq n}{\min}(v_{ki})\leq v_{ki}\leq v_{ki}^{o}\\ 1-\frac{v_{ki}^{0}-v_{ki}}{\max\left[v_{ki}^{0}-\underset{1\leq i\leq n}{\min}(v_{ki}),\underset{1\leq i\leq n}{\max}(v_{ki})-v_{ki}^{0}\right]},v_{ki}^{0}\leq v_{ki}\leq \underset{1\leq i\leq n}{\max}(v_{ki}) \end{array}$$

### 5.3. Weight of Evaluation Factors

AHP model was developed by Satty [28]; it includes both qualitative and quantitative indicators, and ranks programs based on multiple criteria. It has been widely used in macro (complex and realistic) and human (management subjective) problems [29,30,31]. Nowadays, it is used in many research fields [32,33,34,35]. 

In this study, the low-carbon economy system includes four levels (Table 1).

If $a_{ij}$ is the result of comparing *i* with *j.* The matrix is

(9) $$A=\left[\begin{array}{cccc} a_{11} & a_{12} & \ldots & a_{1n}\\ a_{21} & a_{22} & \ldots & a_{2n}\\ \ldots & \ldots & \ldots & \ldots \\ a_{n1} & a_{n2} & \ldots & a_{nn} \end{array}\right]$$

We employed the Delphi method to analyze the relative importance of D, P, S and R. In this study, we provided a scoring table for 30 experts from the universities, scientific institutions and government decision-making departments. Then we calculated the judgment matrix, obtained the indicator weighting and established pair-wise comparison matrix for A (Table 5).Finally, we found that “State” (S) was crucial to the low-carbon economy level, and it was the base for low-carbon economy, Therefore, weight of S was the highest. 

$m_{i}$ is the product of every row:

(10) $$m_{i}=\prod_{i=1}^{4}a_{ij},\;(i=1,\;2,\;3,\;4)$$

(11) $$\bar{w_{i}}=\sqrt[{4}]{m_{i}};\;\bar{w}={\left(\bar{w_{1}},\bar{w_{2}},\ldots ,\bar{w_{n}}\right)}^{T}$$

(12) $$w_{i}=\frac{\bar{w_{i}}}{\sum_{i=1}^{n}\bar{w_{i}}},\;(i=1,\;2,\;3,\;4);\;\lambda_{\max}=\frac{1}{n}\sum_{i=1}^{n}\frac{{\left(AW\right)}_{i}}{w_{i}}$$

(13) $$AW=\left[\begin{array}{cccc} a_{11} & a_{12} & \ldots & a_{1n}\\ a_{21} & a_{22} & \ldots & a_{2n}\\ \ldots & \ldots & \ldots & \ldots \\ a_{n1} & a_{n2} & \ldots & a_{nn} \end{array}\right]\times \left|\begin{array}{c} w_{1}\\ w_{2}\\ •\\ w_{j} \end{array}\right|$$

Finally, we calculated the weight of D, P, S and R were 0.1186, 0.2162, 0.4141 and 0.2511. The eigenvalue $\lambda_{\max}$ = 4.1576.

Then, we tested CI (the consistency index) = 0.0525. CR (the consistency ratio) = 0.059 ≤ 0.10.

(14) $$CI=\frac{\lambda_{\max}-n}{n-1}\;CR=\frac{CI}{RI}$$

*RI* is used to modify the *CI*. In this paper, *RI* = 0.90.

### 5.4. The synthetic Index and Sub-Index of the Low-Carbon Economy

We calculated the indexes according to the aforesaid principles, then used a composite index that contains rule hierarchy composite index and comprehensive composite index (using the hundred percentage point system). In other words, the method of linear weighted sum is used to calculate index value in all rule hierarchies, and then from its result the low-carbon evaluation index (LCEI) value is obtained. 

We use $z_{j}$ to express the numerical value of second grades in the comprehensive system; in order, there are D index, P index, S index and R index. Then we set $x_{k}$ as the dimensionless index after normalization and the $w_{k}$ as the weight of the indexes (Table 1).

(15) $$z_{j}=\sum x_{k}w_{k}\;(j=D,P,R,S);\;\;LCEI=z_{D}+z_{P}+z_{S}+z_{R}$$

We present the low-carbon economy level of China’s 30 regions in Table 6. 

## 6. Assessing Coupling Coordination between the Sub-Indexes

As mentioned above, the horizontal comparisons of the coordination degrees are shown in Table 7, the coordination degrees of the 30 regions in China were calculated and analyzed and were based on the model of coordination degree in 2015.

The coupling degree (C) results show a pattern for that central area lower than the west area and the west area lower than the east area. The average degree of the whole country is 0.6723, which is higher than west and central areas but lower than the east area. 

The coordination degree (D) results show a pattern for the west area lower than the central area and the central area lower than the east area. The average degree of the whole country is 0.5840, which is higher than the west and central areas but lower than the east area.

Moreover, based on the model of coordination degree in 2015, the coordination degrees of the 30 regions in China were calculated and analyzed.

The results indicated the four sub systems were primary coordination in the east area and bare coordination in the central and west areas in 2015. It indicated that four sub-indexes should be developed at the same pace and be improved based on stable development. The construction of low-carbon economy in mid-west areas is the key in China.

## 7. Results and Analysis

### 7.1. Results

In Table 8, the numerical value increases gradually; all provinces, municipalities and autonomous regions show varying increasing rates; the level of low-carbon economy varies by province. The index level shows a gradually decreasing trend from southeastern coastal areas to the north on spatial variation.

**Figure 2:**
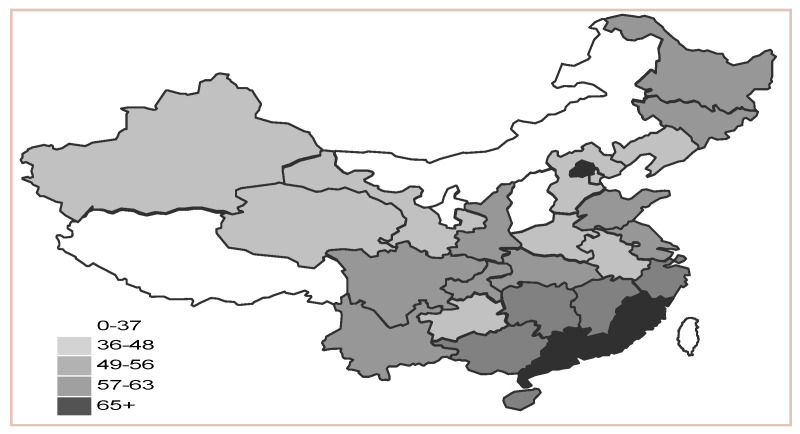
Cluster analysis map of low-carbon economy level of China.

The Figure 2 reflects the distribution of development capability of regional low-carbon economy in China in 2015. It can be seen from Figure 2 that in the spatial distribution, the numerical value of development capability shows a pattern that indicates the southeast is gradually higher than the north. While the clear winner would be the east, which overall stands on the top. This spatial distributive characteristic is closely related to China’s industrial structure, economic development and resource distribution features. For example, Shanxi and Inner Mongolia are important coal-producing bases where coal mines and resources are so crowded that they have weak low-carbon economy development. However, Guangdong and Fujian relatively have lack of natural resources so that their economy has reached a higher level, and the low-carbon economy develops well. Although Beijing is located in northern China and is very near the coal resource centers geographically, with coal making up such a large proportion in its energy structure, it has a well-developed economy; especially, the proportion of its tertiary industry is over 80%. Its industry is mainly based on modern manufacturing and tech industry; therefore, Beijing is under less pressure on low-carbon economy and It ranks 1st in low-carbon economy. However, industrial manufacturing still accounts for a definite proportion of the industrial structure in Shanghai; as a result, Shanghai is under more pressure on low-carbon economy than Beijing even though its economy is better.

In order to reveal the figures of low-carbon economy in different regions, we used Ward’s method to do cluster analysis on China’s 30 regions based on SPSS statistical software. For instance, the results of 2015 were shown in Table 8.

We found that the 30 regions can be divided into 4 categories. These 4 categories correspond respectively to the 4 statuses: leading status, good status, medium status and poor status. The evaluation results show that the numerical value varies greatly in different regions (Table 9). The leading status is almost all in the east area except Jiangxi, Guangxi and Hunan, which are included in the central area. Shanxi, Inner Mongolia and Ningxia fall into poor status.

**Figure 3:**
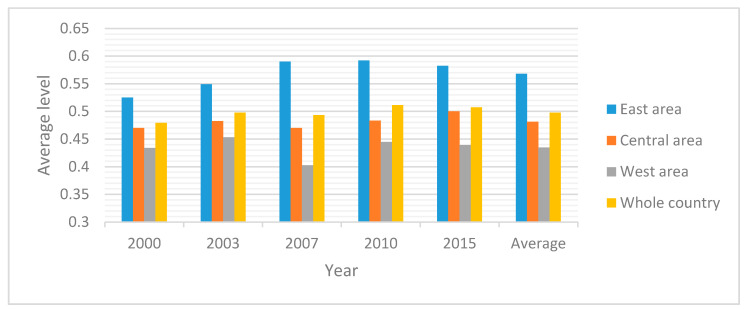
Average low-carbon economy level of China and its three areas.

**Figure 4:**
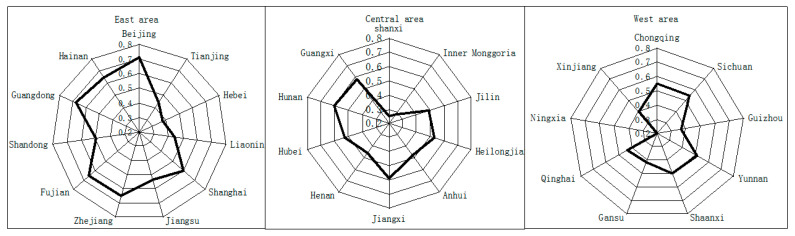
Comparison of the average low-carbon economy level of the three areas.

Figure 3 and Figure 4 compare the difference of average low-carbon economy level in different regions. They show that the numerical value of east regional low-carbon economy shows a pattern that is gradually higher than the west region. The numerical value of low carbon economic development in the south region is higher than the north region by degrees. Almost all parts of the west are low level. Ningxia is considered to have very low level. We find that the average level has shown a downward trend since 2010. This is due to the decline in economic growth and GDP. Growth rate was 10.3% in 2010 and 7.4% in 2015.

### 7.2. Analysis of DPSR Sub-Indexes

In view of the driving sub-index, state sub-index and pressure sub-index, there are some differences in all regions, and the leading status is significantly better than the poor status. As seen from the “Response” sub-index, the resource consumption and degree of pollution vary from province to province, as well as pollution abatement and efforts for environmental protection. All these factors reflect that the more efforts the government, system, society and individual make to improve the status of resources, the better the low-carbon economy develops.

**Figure 5:**
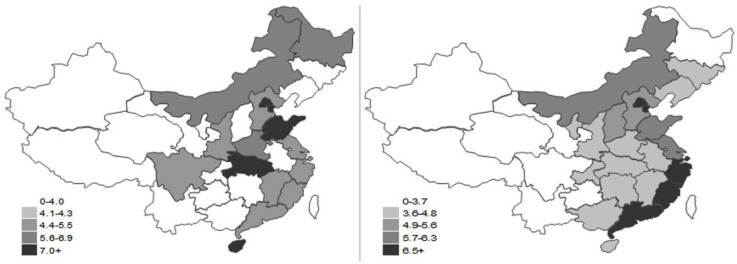
Map of drive sub-index in 2000 and 2015.

The drive sub-index varies greatly from province to province. It shows a pattern with the indication of northeast gradually lower than southeast, which is determined by China’s terrains and regional development policies. From Figure 5, we can see that the regions with high grades of drive sub-index value are all located in coastal areas. This is because these areas have significant regional advantages, which make the input cost of commerce activities and production activities much lower than in other regions. Therefore, their input and output efficiency in economic activities is relatively high. In addition to that, in the earlier stage of reform and opening, China has given special importance to the economic development in coastal areas by providing support in policies. Various regional preferential policies were introduced to form advantages for development; thus, their level of driving force on low-carbon economy is higher than the average level in China.

**Figure 6:**
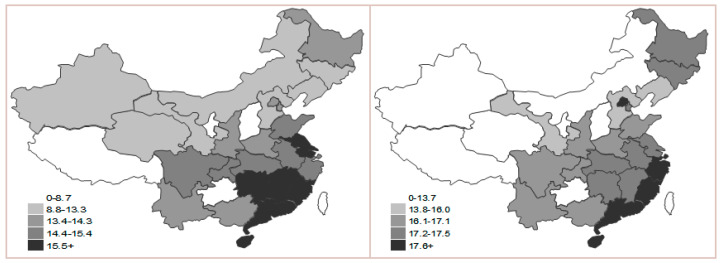
Map of pressure sub-index in 2000 and 2015.

The situation of the pressure sub-index generally indicates the north being gradually lower than the south, the west gradually lower than the east, and hopping in some areas as shown in (Figure 6), which is determined by various causes of development foundations, regional conditions and development policies in all regions. For quite a long time, the speed of economic development in China’s eastern coastal areas was much faster than in central and western areas. In addition, the coming years offer a critical period for western areas to accelerate industrialization; thus, the low-carbon pressure in east is relatively lower than in the west. We can conclude that the index value in the rule hierarchy of pressure has gradually decreasing effects around the main energy supplement region and the surrounding areas, so Shanxi, Inner Mongolia, Ningxia and Qinghai have the most pressure on low-carbon economy.

**Figure 7:**
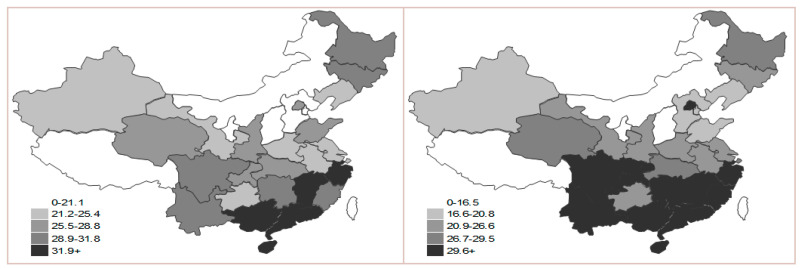
Map of state sub-index in 2000 and 2015.

The situation of the state sub-index indicates the north being gradually lower than the south; therefore, the ranks of Hainan, Guangxi and Fujian are relatively higher, while Shaannxi, Hunan and Hubei are in the second tier (Figure 7). This is because southeastern coastal areas have good economic development with external supply of energy, oil and natural gas. Hydroelectricity and nuclear electricity occupy a large proportion in the structure of energy consumption, so the low-carbon economy in these regions is in good status. However, although central and southern areas have certain coal resources, the consumption far outstrips the production. As a result, the external supply of energy is badly needed. At the same time, these areas that are rich in forest resources vigorously develop hydroelectricity and pursue rational use of energy, so the numerical value in these regions is better than it is in the north.

**Figure 8:**
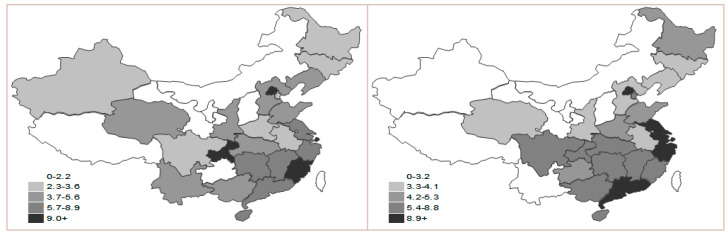
Map of response sub-index in 2000 and 2015.

The situation of the response sub-index shows a pattern of hopping and overlapping in some areas. Beijing, Shanghai and Guangdong rank the first three in 2015, while in the north, northwest and southeast, the index values are gradually low (Figure 8). This is due to the combined impact of the economic and scientific development level, energy consumption efficiency and industrial structure. According to the statistical yearbook, major consumption of energy in west regions is from coal, and there are more coal resources without desulfurization, which combines with low economic development and inadequate input in environment protection. As a result, the efficiency of energy process and conversion is low, and the productivity of carbon falls behind.

### 7.3. Analysis of the Degree of Coordination among the Four Sub-Indexes

As mentioned above, the horizontal comparisons of the coordination degrees are shown in Table 8; the coordination degrees of the 30 regions in China were calculated and analyzed and were based on the model of coordination degree in 2015.

The coupling degree (C) results show a pattern for the central area lower than the west area and the west area lower than the east area. The average degree of the whole country is 0.6723, which is higher than the west and central areas but lower than the east area. 

The coordination degree (D) results show a pattern for the west area lower than the central area and the central area lower than the east area. The average degree of the whole country is 0.5840, which is higher than the west and central areas but lower than the east area.

Moreover, based on the model of coordination degree in 2015, the coordination degrees of the 30 regions in China were calculated and analyzed. 

The results indicated the four sub-systems were primary coordination in the east area and bare coordination in the central and west areas in 2015.In addition, four sub-indexes should be developed at the same pace and improved on the basis of stable development. It shows that promoting the development of a low-carbon economy in mid-west areas is the key in China.

## 8. Conclusions

Based on the above results, the following conclusions are drawn:

All 30 regions show varying increasing rates. The regional distributions of the sub-indexes are the combined impacts of regional economic development level, resource endowment and policy support. The numerical values in China’s 30 regions vary greatly; as a whole, they indicates the north is gradually lower than the southeastern coastal areas, and the east is higher than the west; the south is higher than the north, and there is overlapping in some areas. Beijing and Shanghai are far ahead while Shanxi, Inner Mongolia and Ningxia are backward.

As compared to the developed countries that have already achieved industrialization, China focuses particularly on achieving sustainable development by combining industrialization with ecological civilization. It is worth noting that China has large regional differences. Compared with the developed regions in the east, there are still a lot of heavy-duty industries in the central and western regions. Industrial structure transformation is more difficult. Therefore, promoting the development of low-carbon economy in mid-west areas is the key in China.

The improvement of people’s living quality and social welfare can best embody the development of low-carbon economy. The result of this study may be helpful for policy makers to formulate effective policies and take actions in the future.

We firmly believe that ecological civilization construction, industrialization and urbanization are not mutually exclusive at all. We must emphasize realizing sustainable development through the low-carbon system while people’s material life has benefited a lot. Therefore, we ought to change the bad social consumption mode, as well as wrong consuming concepts and patterns. Besides, we must make great efforts to turn the low-carbon way of life into a conscious behavior while improving our quality of life and stick to it in concrete activities.

## Figures and Tables

**Table 1:** Index system of low-carbon economy.

First Grade	Second Grade	Weight	Third Grade	Weight	Fourth Grade	Weight	Positive or Negative
Comprehensive level of low-carbon economy (A)	Driver (D)	0.1186	Driver for social development (D1)	0.0593	Natural Population Growth (D11, ‰)	0.0272	Moderate
					Urbanization Rate (D12, %)	0.0247	Moderate
					Engel’s coefficient on urban and rural households (D13, %)	0.0075	Negative
			Driver for economic development (D2)	0.0593	per capita GDP (D21, yuan per capita)	0.0183	Positive
					GDP growth rate (D22, %)	0.0065	Positive
					The average income of rural and urban family (D23, yuan per capita)	0.0345	Positive
	Pressure (P)	0.2162	resource pressure (P1)	0.0865	The energy consumption per unit of GDP (P11, tons of coal per 10,000 yuan)	0.0605	Negative
					Electricity consumption per capita (P12, kwh per capita)	0.0259	Negative
			environmental pressure (P2)	0.1297	The industrial waste-gas discharge per unit of GDP (P21, cubic meter per 10,000 yuan)	0.0741	Negative
					SO_2_ emission per unit of GDP (P22, ton per 10,000 yuan)	0.0371	Negative
					Public transportations per 10,000 people (P23)	0.0185	Positive
	Status (S)	0.4141	Status of low-carbon consumption (S1)	0.2071	Carbon emissions per capita (S11, ton per capita)	0.1305	Negative
					The carbon emissions of residents’ consumption (S12, ton per 10,000 yuan)	0.0541	Negative
					The carbon emissions of government consumption (S13, ton per 10,000 yuan)	0.0225	Negative
			Status of low-carbon resources (S2)	0.2071	Proportion of consumption of raw coal (S21, %)	0.0941	Negative
					Carbon emissions per unit of energy (S22, ton of CO_2_ per ton of coal)	0.0188	Negative
					Forest coverage (S23, %)	0.0941	Positive
	Response (R)	0.2511	Scientific Response (R1)	0.1758	Expenditure on science and technology per capita (R11, yuan per capita)	0.0189	Positive
					The efficiency of energy process and conversion (R12, %)	0.0181	Positive
					Carbon productivity (R13, 10,000 yuan per ton)	0.1387	Positive
			Policy Response (R2)	0.0753	The proportion the tertiary industry output value accounts for GDP (R21, %)	0.0442	Positive
					The development plan of low-carbon economy (R22)	0.0077	Positive
					Policy of carbon tax (R23)	0.0081	Positive
					Supervision and statistics system of carbon emissions (R24)	0.0154	Positive

**Table 2:** Classification of the coupling coordinated degree.

Coordination Degree	Coordination Level	Coordination Degree	Coordination Level
0.01 < CD2 ≤ 0.10	Extreme disorder ≤	0.51 < CD2 ≤ 0.60	Bare coordination
0.11 <CD2 ≤ 0.20	Serious disorder	0.61 < CD2 ≤ 0.70	Primary coordination
0.21 <CD2 ≤ 0.30	Moderate disorder	0.71 < CD2 ≤0.80	Intermediate coordination
0.31 <CD2 ≤ 0.40	Mild disorder	0.81 < CD2 ≤0.90	Favorable coordination
0.41 <CD2 ≤ 0.50	On the verge of disorder	0.91 < CD2 ≤ 0.10	Quality coordination

**Table 3:** Coefficient of CO_2_ emissions in China.

Energy Type	A		B	C = A × B × (44 / 12) × 1000		D = C × 4186.8 × 10−9 × 10−3		E		F = D × E	
	IPCC (2006)		Oxidation Rate in the Combustion	IPCC (2006)		Original Coefficient		Calorific Value of Unit Fuel in China		Suggested Coefficient	
	Carbon Emission Coefficient	Measurement		CO_2_ Emission Coefficient	Measurement	Original Coefficient	Measurement	Average Calorific Value of Unit Fuel in China	Measurement	Coefficient	Measurement
Crude coal	25.8	kgC/GJ	1	94,600	kgCO_2_/TJ	0.000396071	kgCO_2_/Kcal	5000	Kcal/Kg	1.98	kgCO_2_/Kg
Coke	29.2	kgC/GJ	1	107,066.67	kgCO_2_/TJ	0.000448267	kgCO_2_/Kcal	6800	Kcal/Kg	3.05	kgCO_2_/Kg
Crude oil	20	kgC/GJ	1	73,333.33	kgCO_2_/TJ	0.000307032	kgCO_2_/Kcal	10,000	Kcal/Kg	3.07	kgCO_2_/Kg
Gasoline	18.9	kgC/GJ	1	69,300	kgCO_2_/TJ	0.000290145	kgCO_2_/Kcal	10,300	Kcal/Kg	2.99	kgCO_2_/Kg
Jet kerosene	19.6	kgC/GJ	1	71,866.67	kgCO_2_/TJ	0.000300891	kgCO_2_/Kcal	10,300	Kcal/Kg	3.10	kgCO_2_/Kg
Diesel oil	20.2	kgC/GJ	1	74,066.67	kgCO_2_/TJ	0.000310102	kgCO_2_/Kcal	10,200	Kcal/Kg	3.16	kgCO_2_/Kg
Fuel oil	21.1	kgC/GJ	1	77,366.67	kgCO_2_/TJ	0.000323919	kgCO_2_/Kcal	10,000	Kcal/Kg	3.24	kgCO_2_/Kg
Refinery gas	16.8	kgC/GJ	1	61,600	kgCO_2_/TJ	0.000257907	kgCO_2_/Kcal	11,000	Kcal/Kg	2.84	kgCO_2_/Kg
Natural gas	15.3	kgC/GJ	1	56,100	kgCO_2_/TJ	0.000234879	kgCO_2_/Kcal	9310	kgCO_2_/M3	2.19	kgCO_2_/M3
Liquefied petroleum gas	17.2	kgC/GJ	1	63,066.67	kgCO_2_/TJ	0.000264048	kgCO_2_/Kcal	12,000	kgCO_2_/M3	3.17	kgCO_2_/M3

**Table 4:** Energy consumption and CO_2_ emissions.

Year	Crude Coal Consumption (104 Tons)	Coke Consumption (104 Tons)	Crude Oil Consumption (104 Tons)	Gasoline Oil Consumption (104 Tons)	Jet Kerosene Consumption (104 Tons)	Diesel Oil Consumption (104 Tons)	Fuel Oil Consumption (104 Tons)	Natural Gas Consumption (104 Tons)	CO_2_ Emissions
1995	137,677	10,725.3	14,886.4	2910	512.1	4321	3693.7	177.4	49.46
1996	144,734.46	11,865	15,865.01	3182.4	555.5	4825.14	3632.31	185.9	51.56
1997	139,248.26	10,927	17,367.2	3312	681.71	5291.21	3848.2	196.44	51.58
1998	129,492.2	11,447.82	17,395.31	3328.6	706.41	5309.7	3828.6	202.6	49.77
1999	139,336.5	10,460.52	18,949.5	3389.3	824.21	6231.63	3934.11	214.94	52.58
2000	135,690	10,840.8	21,232	3505	871.6	6578.57	3872.8	245	52.64
2001	143,063	11,931.5	21,410.74	3597.6	890.3	6917.58	3850.22	274.3	54.55
2002	153,585	12,803.12	22,694.1	3804.32	919.2	7560.87	3723.9	292	57.16
2003	183,760	15,926.5	25,180.72	4118.52	921.61	8498.16	4330.34	339.1	67.21
2004	212,162	18,067.01	29,009.31	4695.72	1060.9	10,072.94	4844.8	397	77.04
2005	243,375	25,105.8	30,088.9	4855	1076.8	10,889.42	4244.2	466.1	84.06
2006	270,639	28,298	32,245.2	5242.55	1124.74	11,652.71	4471.2	573.33	92.20
2007	290,410	31,168.12	34,031.6	5519.1	1243.72	12,420.25	4157.5	705.23	96.89
2008	300,605	32,120.23	35,510.34	6145.52	1294.01	13,475.46	3236.8	813	97.20
2009	325,003	36,350	38,128.59	6172.69	1450.49	13,494.83	2828.8	895.2	102.87
2010	349,008	38,702.8	42,874.6	6956	1765.2	14,655.17	3758	1080.2	113.50
2011	388,961	42,063.3	43,965.8	7596	1816.7	15,593.54	3662.8	1341.1	122.97
2012	411,727	44,805.2	46,678.9	8166	1956.6	16,900.67	3683.3	1497	129.84
2013	424,426	45,851.9	48,652.2	9366	2164.1	17,106.75	3954	1705.4	134.65
2014	411,613	46,884.9	51,547	9776	2335.4	17,127.02	4400.5	1868.9	134.94
2015	397,014	44,058.7	54,088.3	11,368	2663.7	17,280.44	4662	1931.7	133.44
2016	384,560	45,462.4	56,025.9	11,866	2970.7	16,736.39	4631	2078.1	131.97

**Table 5:** Pair-wise comparison matrix for A.

A	D	P	S	R
D	1	1/4	1/7	1/5
P	4	1	1/4	1/2
S	7	4	1	4
R	5	2	1/4	1

**Table 6:** Low-carbon economy level in China.

Regions and Areas		2000	2003	2007	2010	2015	Average
East area	Beijing	0.5882	0.6603	0.7980	0.7829	0.8634	0.7386
	Tianjin	0.3411	0.3505	0.5329	0.5340	0.5367	0.4590
	Hebei	0.3783	0.3715	0.3581	0.3881	0.4660	0.3924
	Liaoning	0.4309	0.4731	0.4136	0.4341	0.4443	0.4392
	Shanghai	0.5259	0.5750	0.6332	0.6509	0.5744	0.5919
	Jiangsu	0.4942	0.5364	0.5451	0.5581	0.5613	0.5390
	Zhejiang	0.6057	0.6564	0.6469	0.6721	0.6414	0.6445
	Fujian	0.6265	0.6241	0.6821	0.6832	0.6345	0.6501
	Shandong	0.5299	0.5250	0.4755	0.4623	0.4724	0.4930
	Guangdong	0.6256	0.6393	0.7191	0.7179	0.6618	0.6727
	Hainan	0.6299	0.6274	0.6862	0.6307	0.5528	0.6254
	Average	0.5251	0.5490	0.5901	0.5922	0.5826	0.5678
Central area	Shanxi	0.2756	0.2484	0.2420	0.2315	0.2481	0.2491
	Inner Mongolia	0.3232	0.3058	0.2181	0.2437	0.2763	0.2734
	Jilin	0.5001	0.4981	0.4843	0.4932	0.5051	0.4962
	Heilongjiang	0.5075	0.5535	0.5469	0.5176	0.5312	0.5313
	Anhui	0.4491	0.4673	0.4833	0.4943	0.5580	0.4904
	Jiangxi	0.5635	0.5855	0.5913	0.6032	0.5998	0.5887
	Henan	0.4452	0.4812	0.4452	0.4623	0.5291	0.4726
	Hubei	0.4957	0.5213	0.5254	0.5597	0.5741	0.5352
	Hunan	0.5963	0.6012	0.5755	0.6115	0.6025	0.5974
	Guangxi	0.5446	0.5641	0.5897	0.6170	0.5758	0.5782
	Average	0.4701	0.4826	0.4702	0.4834	0.5000	0.4813
West area	Chongqing	0.5936	0.6194	0.4067	0.5733	0.5760	0.5538
	Sichuan	0.5045	0.5237	0.5419	0.5956	0.5657	0.5463
	Guizhou	0.3203	0.3694	0.3351	0.4159	0.5176	0.3917
	Yunnan	0.4982	0.5059	0.4910	0.5487	0.5369	0.5161
	Shaanxi	0.5032	0.5078	0.5039	0.4795	0.4925	0.4974
	Gansu	0.3910	0.4013	0.4197	0.4389	0.4422	0.4186
	Qinghai	0.4259	0.4622	0.3845	0.4671	0.3813	0.4242
	Ningxia	0.2546	0.2366	0.1530	0.1505	0.1931	0.1976
	Xinjiang	0.4137	0.4530	0.3866	0.3323	0.2478	0.3667
	Average	0.4339	0.4533	0.4025	0.4447	0.4392	0.4347
Whole country	Average	0.4791	0.4979	0.4933	0.5112	0.5073	0.4978

**Table 7:** The cluster results.

Category one (Leading status)	Beijing, Guangdong, Hainan, Fujian, Zhejiang, Shanghai, Jiangxi, Guangxi, Hunan
Category two (Good status)	Heilongjiang, Sichuan, Jiangsu, Tianjin, Hubei, Shaanxi, Yunnan, Jilin, Anhui, Shandong
Category three (Medium status)	Chongqing, Henan, Gansu, Liaoning, Xinjiang, Qinghai, Hebei, Guizhou
Category four (Poor status)	Shanxi, Inner Mongolia, Ningxia

**Table 8:** The regional comparisons in 2015.

Regions and Areas		d(x)	p(y)	s(z)	p(k)	C	T	D	Coordination Level
East area	Beijing	0.0897	0.2108	0.3488	0.2142	0.8980	0.8634	0.8805	Favorable coordination
	Tianjin	0.0696	0.1959	0.2401	0.0311	0.7486	0.5367	0.6338	Primary coordination
	Hebei	0.0468	0.1429	0.2593	0.0169	0.6314	0.4660	0.5424	Bare coordination
	Liaoning	0.0411	0.1494	0.2279	0.0259	0.6984	0.4443	0.5570	Bare coordination
	Shanghai	0.0847	0.1795	0.2541	0.0562	0.8451	0.5744	0.6968	Primary coordination
	Jiangsu	0.0668	0.1920	0.2719	0.0306	0.7241	0.5613	0.6375	Primary coordination
	Zhejiang	0.0777	0.1838	0.3493	0.0306	0.6933	0.6414	0.6668	Primary coordination
	Fujian	0.0696	0.1850	0.3619	0.0181	0.6038	0.6345	0.6189	Primary coordination
	Shandong	0.0589	0.1756	0.2203	0.0176	0.6740	0.4724	0.5643	Bare coordination
	Guangdong	0.0820	0.1906	0.3698	0.0194	0.6219	0.6618	0.6415	Primary coordination
	Hainan	0.0346	0.1789	0.3071	0.0322	0.6402	0.5528	0.5949	Bare coordination
	Average	0.0656	0.1804	0.2918	0.0448	0.7656	0.5826	0.6679	Primary coordination
Central area	Shanxi	0.0427	0.0841	0.0906	0.0307	0.9064	0.2481	0.4742	On the verge of disorder
	Inner Mongolia	0.0566	0.0990	0.1145	0.0061	0.6442	0.2763	0.4219	On the verge of disorder
	Jilin	0.0313	0.1632	0.2972	0.0134	0.5316	0.5051	0.5182	Bare coordination
	Heilongjiang	0.0293	0.1830	0.2925	0.0264	0.6043	0.5312	0.5666	Bare coordination
	Anhui	0.0450	0.1733	0.3249	0.0148	0.5606	0.5580	0.5593	Bare coordination
	Jiangxi	0.0389	0.1838	0.3677	0.0095	0.4707	0.5998	0.5314	Bare coordination
	Henan	0.0381	0.1792	0.2958	0.0160	0.5700	0.5291	0.5491	Bare coordination
	Hubei	0.0485	0.1777	0.3334	0.0145	0.5601	0.5741	0.5670	Bare coordination
	Hunan	0.0491	0.1802	0.3542	0.0190	0.5835	0.6025	0.5929	Bare coordination
	Guangxi	0.0350	0.1713	0.3633	0.0062	0.4215	0.5758	0.4926	On the verge of disorder
	Average	0.0414	0.1595	0.2834	0.0157	0.5888	0.5000	0.5426	Bare coordination
West area	Chongqing	0.0479	0.1764	0.3335	0.0181	0.5873	0.5760	0.5816	Bare coordination
	Sichuan	0.0293	0.1807	0.3409	0.0148	0.5081	0.5657	0.5361	Bare coordination
	Guizhou	0.0357	0.1626	0.2938	0.0255	0.6276	0.5176	0.5700	Bare coordination
	Yunnan	0.0353	0.1332	0.3596	0.0088	0.4620	0.5369	0.4980	On the verge of disorder
	Shaanxi	0.0391	0.1683	0.2649	0.0201	0.6254	0.4925	0.5550	Bare coordination
	Gansu	0.0355	0.1268	0.2547	0.0251	0.6628	0.4422	0.5414	Bare coordination
	Qinghai	0.0344	0.0809	0.2607	0.0052	0.4634	0.3813	0.4203	On the verge of disorder
	Ningxia	0.0371	0.0453	0.0951	0.0157	0.8236	0.1931	0.3988	Mild disorder
	Xinjiang	0.0239	0.0725	0.1354	0.0160	0.7105	0.2478	0.4196	On the verge of disorder
	Average	0.0354	0.1274	0.2599	0.0166	0.6046	0.4392	0.5153	Bare coordination
Whole country	Average	0.0475	0.1558	0.2784	0.0257	0.6723	0.5073	0.5840	Bare coordination

**Table 9:** The regional comparisons in 2015.

Regions and Areas		d(x)	p(y)	s(z)	p(k)	C	T	D	Coordination Level
East area	Beijing	0.0897	0.2108	0.3488	0.2142	0.8980	0.8634	0.8805	Favorable coordination
	Tianjin	0.0696	0.1959	0.2401	0.0311	0.7486	0.5367	0.6338	Primary coordination
	Hebei	0.0468	0.1429	0.2593	0.0169	0.6314	0.4660	0.5424	Bare coordination
	Liaoning	0.0411	0.1494	0.2279	0.0259	0.6984	0.4443	0.5570	Bare coordination
	Shanghai	0.0847	0.1795	0.2541	0.0562	0.8451	0.5744	0.6968	Primary coordination
	Jiangsu	0.0668	0.1920	0.2719	0.0306	0.7241	0.5613	0.6375	Primary coordination
	Zhejiang	0.0777	0.1838	0.3493	0.0306	0.6933	0.6414	0.6668	Primary coordination
	Fujian	0.0696	0.1850	0.3619	0.0181	0.6038	0.6345	0.6189	Primary coordination
	Shandong	0.0589	0.1756	0.2203	0.0176	0.6740	0.4724	0.5643	Bare coordination
	Guangdong	0.0820	0.1906	0.3698	0.0194	0.6219	0.6618	0.6415	Primary coordination
	Hainan	0.0346	0.1789	0.3071	0.0322	0.6402	0.5528	0.5949	Bare coordination
	Average	0.0656	0.1804	0.2918	0.0448	0.7656	0.5826	0.6679	Primary coordination
Central area	Shanxi	0.0427	0.0841	0.0906	0.0307	0.9064	0.2481	0.4742	On the verge of disorder
	Inner Mongolia	0.0566	0.0990	0.1145	0.0061	0.6442	0.2763	0.4219	On the verge of disorder
	Jilin	0.0313	0.1632	0.2972	0.0134	0.5316	0.5051	0.5182	Bare coordination
	Heilongjiang	0.0293	0.1830	0.2925	0.0264	0.6043	0.5312	0.5666	Bare coordination
	Anhui	0.0450	0.1733	0.3249	0.0148	0.5606	0.5580	0.5593	Bare coordination
	Jiangxi	0.0389	0.1838	0.3677	0.0095	0.4707	0.5998	0.5314	Bare coordination
	Henan	0.0381	0.1792	0.2958	0.0160	0.5700	0.5291	0.5491	Bare coordination
	Hubei	0.0485	0.1777	0.3334	0.0145	0.5601	0.5741	0.5670	Bare coordination
	Hunan	0.0491	0.1802	0.3542	0.0190	0.5835	0.6025	0.5929	Bare coordination
	Guangxi	0.0350	0.1713	0.3633	0.0062	0.4215	0.5758	0.4926	On the verge of disorder
	Average	0.0414	0.1595	0.2834	0.0157	0.5888	0.5000	0.5426	Bare coordination
West area	Chongqing	0.0479	0.1764	0.3335	0.0181	0.5873	0.5760	0.5816	Bare coordination
	Sichuan	0.0293	0.1807	0.3409	0.0148	0.5081	0.5657	0.5361	Bare coordination
	Guizhou	0.0357	0.1626	0.2938	0.0255	0.6276	0.5176	0.5700	Bare coordination
	Yunnan	0.0353	0.1332	0.3596	0.0088	0.4620	0.5369	0.4980	On the verge of disorder
	Shaanxi	0.0391	0.1683	0.2649	0.0201	0.6254	0.4925	0.5550	Bare coordination
	Gansu	0.0355	0.1268	0.2547	0.0251	0.6628	0.4422	0.5414	Bare coordination
	Qinghai	0.0344	0.0809	0.2607	0.0052	0.4634	0.3813	0.4203	On the verge of disorder
	Ningxia	0.0371	0.0453	0.0951	0.0157	0.8236	0.1931	0.3988	Mild disorder
	Xinjiang	0.0239	0.0725	0.1354	0.0160	0.7105	0.2478	0.4196	On the verge of disorder
	Average	0.0354	0.1274	0.2599	0.0166	0.6046	0.4392	0.5153	Bare coordination
Whole country	Average	0.0475	0.1558	0.2784	0.0257	0.6723	0.5073	0.5840	Bare coordination
